# Evaluating the influence of the obesity paradox on survival outcomes in patients being treated surgically for rectal cancer—a systematic review and meta-analysis

**DOI:** 10.1007/s00384-025-04957-z

**Published:** 2025-08-18

**Authors:** Matthew G. Davey, Noel E. Donlon, Mark Donnelly, Eanna J. Ryan, Odhran K. Ryan, Ian S. Reynolds, William P. Joyce

**Affiliations:** 1 Royal College of Surgeons in Ireland, 123 St. Stephens Green, Dublin 2, Dublin, Ireland; 2 Department of Surgery, Galway Clinic, Doughiska, Galway, Republic of Ireland

**Keywords:** Obesity, Rectal cancer, Obesity surgery, Body mass indices

## Abstract

**Background:**

Obesity is a well-established risk factor for rectal cancer development. The association between obesity and survival outcomes in those undergoing resection for rectal cancer remains unclear. The objective of this study was to perform a systematic review and meta-analysis evaluating the association between obesity and overall (OS) and disease-free (DFS) in patients undergoing surgery for rectal cancer with curative intent.

**Methods:**

A systematic review was performed as per PRISMA guidelines. Descriptive statistics (Fisher’s exact test (†)) were used. Meta-analyses were performed using Mantel–Haenszel and generic inverse variance methods using RevMan version 5.4.

**Results:**

Twenty-three studies with 22,520 patients were included (mean follow-up: 59.7 months). Overall, 18.5% of patients were living with obesity (4174/22,520). Obesity was associated with poorer DFS [60.5% (2289/3783) vs. 62.4% (9576/15,335), *P* = 0.029, †]; however, a non-significant difference was observed at meta-analysis [hazard ratio (HR): 1.12, 95% confidence interval (95% CI) 0.90–1.40, *P* = 0.320, heterogeneity (*I*^2^) = 77%]. Obesity was associated with enhanced OS [67.8% (2500/3687) vs. 59.8% (9048/15,125), *P* < 0.001, †], results which were replicated at meta-analysis [HR 1.24, 95% CI 1.03–1.50, *P* = 0.020, *I*^2^ = 59%]. Using time-to-effect modelling, a non-significant difference in DFS [HR 0.93, 95% CI 0.69–1.24, *P* = 0.600, *I*^2^ = 57%] and OS [HR 1.00, 95% CI 0.73–1.37, *P* = 1.000, *I*^2^ = 69%] was observed at meta-analysis.

**Conclusion:**

Once diagnosed and being treated with curative intent for rectal cancer, patients living with obesity exhibit similar survival outcomes as those living without obesity. This study refutes hypotheses that an ‘obesity paradox’ is protective for survival in patients with rectal cancer. Given the current obesity epidemic, this concept may warrant incorporation into preoperative counselling.

## Introduction

Cancer and obesity are among the greatest global healthcare concerns, with an accelerated incidence of both in recent decades [1, 2]. Obesity is a well-established risk factor for developing malignancy, with data illustrating that obesity-related cancers represent 11.9% and 13.1% of cancer diagnoses in male and female patients, respectively [3]. Notwithstanding these facts, a ‘paradox’ surrounding the relationship between obesity and survival outcomes in those diagnosed with malignancy has been previously described; the ‘obesity paradox’ describes an observation in which patients with increased body mass indices (BMI) who are diagnosed with malignancy possess a propensity to have improved survival and oncological outcomes relative to their counterparts with normal or low BMI [4]. This concept has been supported in historical and contemporary data, including registry data, and has been successfully translated into both the localised and advanced settings with relevance in several cancer subtypes [5–9].

Nevertheless, controversy exists surrounding the accuracy of the ‘obesity paradox’ in the setting of patients being diagnosed and treated for colorectal carcinoma (CRC); recent studies have demonstrated improved overall (OS) and disease-specific (DSS) survival in patients living with obesity who are diagnosed with CRC [10, 11], and these results have subsequently been ratified in other solid organ malignancies [8, 12]. Moreover, a recent meta-analysis of 55,391 patients being treated for CRC demonstrated that those with increased BMI appeared to have improved survival outcomes relative to those of normal BMI, notwithstanding obesity being a renowned predisposing risk factor responsible for CRC development [13].

However, the biomolecular era has facilitated the substratification of CRC into both primary colonic and rectal cancers, respectively, thus rendering the traditional umbrella term ‘CRC’ subject to reasonable scrutiny [14–16]. Moreover, given their differentiation in embryological origin, natural history of disease, and the varying multimodal treatment strategies adopted for these cancers, it is now accepted that primary cancers of the colon and rectum are to be considered distinct disease entities [14–16]. Therefore, the assumption that these previous results have adopted relevance in the setting of neoplasms of the rectum should be challenged, which creates opportunity for another meta-analysis to be performed to ascertain whether obesity is truly protective in the setting of patients diagnosed with rectal cancer. Accordingly, the aim of the current study was to perform a systematic review and meta-analysis evaluating the association between obesity and OS and disease-free (DFS) in patients undergoing surgery for rectal cancer with curative intent.

## Methods

This systematic review was conducted as detailed by the preferred reporting items for systematic reviews and meta-analyses (PRISMA) guidelines [17]. Ethical approval was not required and not sought from the local institutional review board, as this study uses data from previously published resources. All of the listed authors contributed to formulating the study protocol, and it was then prospectively registered with the International Prospective Register of Systematic Reviews (PROSPERO – CRD42024498395) prior to commencing the review.

### Population, intervention, comparison, outcome (PICO) tool

The PICO framework was adopted [18]. Within this, the clinical research question the authors wished to address was:

- Population – Adult patients aged 18 years or older at diagnosis who are diagnosed with rectal cancer and are due to be treated surgically with curative intent,
- Intervention – Any patients diagnosed who were living with obesity,
- Comparison – Any patients diagnosed who were not living with obesity,
- Outcomes – The study outcomes included: DFS (defined as freedom from invasive cancer recurrence or death) and OS (defined as freedom from death) in order to determine the differences in survival between obese and non-obese patients.

### Search strategy

A formal and robust electronic search was performed of the PubMed, Web of Science and Cochrane (CENTRAL) databases on the 18 December 2023 for relevant studies which would be deemed suitable for inclusion in this study. This review was performed by two independent reviewers. In instances or situations when there was differences in opinion, the senior author was consulted and asked to arbitrate. The search was performed under the following headings: (rectal neoplasm[MeSH Terms]) AND (obesity[MeSH Terms]) which were linked by the Boolean operator ‘AND’. Included studies were limited to those published in the English language and of were acceptable irrespective of whether prospective or retrospective in design. Included studies were restricted based on year of publication, with studies published within the previous two decades only being considered for inclusion. The authors pragmatically excluded studies reporting outcomes for less than 20 patients. For retrieved studies, their titles were initially screened, before the abstracts and full texts which were deemed appropriate were reviewed.

### Inclusion and exclusion criteria

During review, articles were considered for inclusion once they met the following inclusion criteria: (1) Clinical studies of both retrospective or prospective design; (2) studies which were performed to compare DFS and OS outcomes in patients who were indicated to undergo surgery for rectal cancer; (3) studies had to compare outcomes in two patient groups (i.e. obese and non-obese patients); (4) studies had to include data from adult patients aged 18 years or older; and (5) studies had to have reported results in the past 20 years (i.e. studies published between and including the years 2004 and 2023). Studies were excluded from this study if they failed to meet the above inclusion criteria.

### Data extraction and quality assessment

As described, the literature search was performed by two independent reviewers (M.G.D and M.D) using the predesigned search strategy, which was agreed on by all authors. Duplicate studies were manually removed by each reviewer. Each reviewer then reviewed the associated titles, abstracts and/or full texts of the retrieved manuscripts to ensure all inclusion criteria were met, before extracting the following data: (1) first author name, (2) year of publication, (3) study design, (4) country of research facility, (5) number of patients indicated to undergo treatment for rectal cancer, (6) number of patients living with and without obesity, (7) clinicopathological parameters, (8) treatment characteristics, (9) survival outcomes (i.e. DFS and OS). Risk of bias and methodological assessment of included studies was undertaken using the Newcastle Ottawa Scale.

### Statistical analysis

Descriptive statistics were deployed to determine the associations between survival outcomes and those living with or without obesity (Fisher’s exact test, †) [19]. Survival outcomes were then expressed as dichotomous outcomes, reported as odds ratios (ORs) with their corresponding 95% confidence interval (CI), following estimation using the Mantel–Haenszel method. Thereafter, the generic inverse variance method was deployed to determine survival outcomes using time-to-effect modelling and expressed as hazard ratios (HRs) with 95% CIs. Assuming that there is a significant degree of heterogeneity between the studies included in this study, random effect models were deployed for all analyses. All tests of significance were two-tailed with *P* < 0.05 indicating statistical significance. Descriptive statistics were performed using the Statistical Package for Social Sciences (SPSS) version 26 (International Business Machines Corporation, Armonk, New York). Meta-analyses were conducted using Review Manager (RevMan), Version 5.4 (Nordic Cochrane Centre, Copenhagen, Denmark).

## Results

### Literature search

**Fig. 1:**
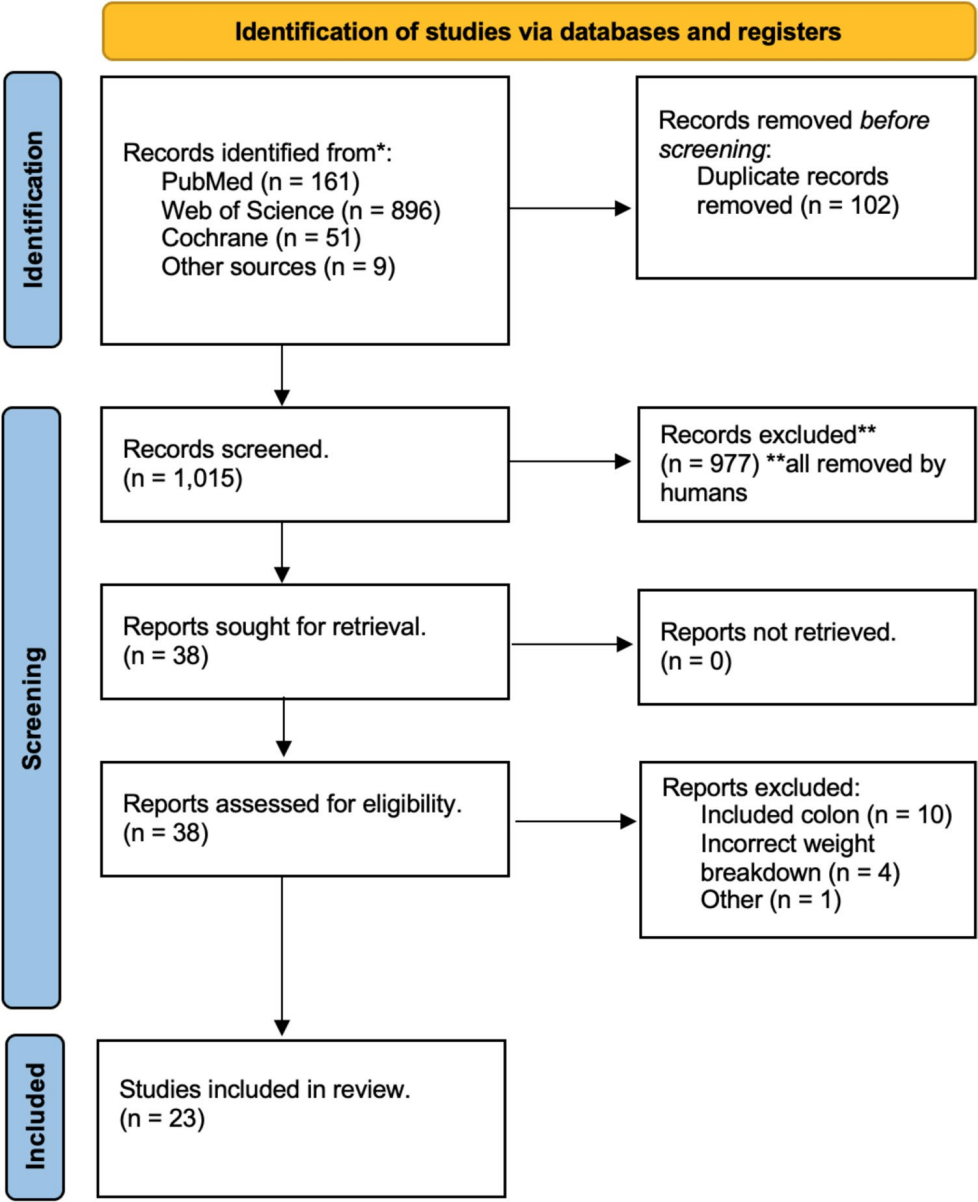
PRISMA flowchart demonstrating the systematic search process

In total, the systematic search strategy identified a total of 1117 studies, of which 102 duplicate studies were manually removed by reviewers. The remaining 1015 studies were screened for relevance, after which 38 had their full texts reviewed. In total, 23 studies met the eligibility criteria and were included in this systematic review and meta-analysis [20–42] (Fig. 1).

### Study characteristics

Of the 23 studies included in this analysis, all studies compared oncological and survival outcomes in patients living with or without obesity who were indicated to undergo treatment for rectal cancer (100.0%, 23/23). In total, there were data included from 19 retrospective cohort studies (82.6%) [5, 20–25, 28–31, 33–36, 38–41], two prospective cohort studies (8.7%) [27, 32], and two prospective, randomised clinical trials (8.7%), respectively [26, 42]. Publication dates of included studies ranged from 2004 to 2023. Study data and respective risk of bias assessments for the 23 included studies are outlined in Table 1.

**Table 1:** Details of the 23 included studies

Title	Year	Study design	Mean age in years (range)	Follow-up (months)	Number	Number obese	Number non-obese	NOS
Aytac	2013	RC	62 (32–94)	120	471	157	314	5
Baird	2019	RC	62 (27–89)	26	220	42	178	6
Ballian	2010	RC	61	-	254	68	186	5
Chern	2010	RC	58	39	586	159	427	6
Clark	2013	RC	61	-	99	41	58	4
Denost	2012	RC	65 (20–90)	-	490	47	443	5
Diefenhardt	2021	RCT	-	33	1265	270	995	7
Gebauer	2017	PC	68 (21–99)	23	9920	1901	8019	8
Gutierrez	2023	RC	66	43	390	59	331	5
Juszczyk	2020	RC	-	38	453	131	322	5
Kalb	2019	RC	65 (18–91)	93	612	127	485	5
Liu	2020	RC	55 (23–82)	46	243	24	219	4
Meyerhardt	2004	RCT	-	120	1688	306	1382	7
Pai	2017	PC	-	-	101	33	68	4
Seishima	2014	RC	61	56	263	64	199	5
Son	2019	RC	-	-	238	119	119	6
Soo Han	2020	RC	59	-	1384	42	1342	6
Sun	2017	RC	55	55	312	63	249	5
Tschann	2023	RC	63	69	106	34	72	3
Xhang	2021	RC	56 (32–80)	-	356	48	308	4
You	2009	RC	63	-	1873	93	1780	7
Zhang	2023	RC	59	69	404	178	226	5
Zhang	2019	RC	59 (22–89)	65	792	168	624	5
			61.1 (18–99)	59.7	22,520	4174	18,346	-

*NOS* Newcastle Ottawa Scale, *RC* retrospective cohort, *RCT* randomised clinical trial, *PC* prospective cohort

### Patient characteristics

In total, data from 22,520 patients was included. The mean age at diagnosis was 61.1 years (range: 19–99). The mean estimated follow-up was 59.7 months. In total, 18.5% of patients were classified as living with obesity at the time of rectal cancer diagnosis (4174/22,520), and 81.5% were classified as not living with obesity (18,346/22,520). A detailed breakdown of data from included studies are outlined in Table 1.

### Disease-free survival

**Fig. 2:**
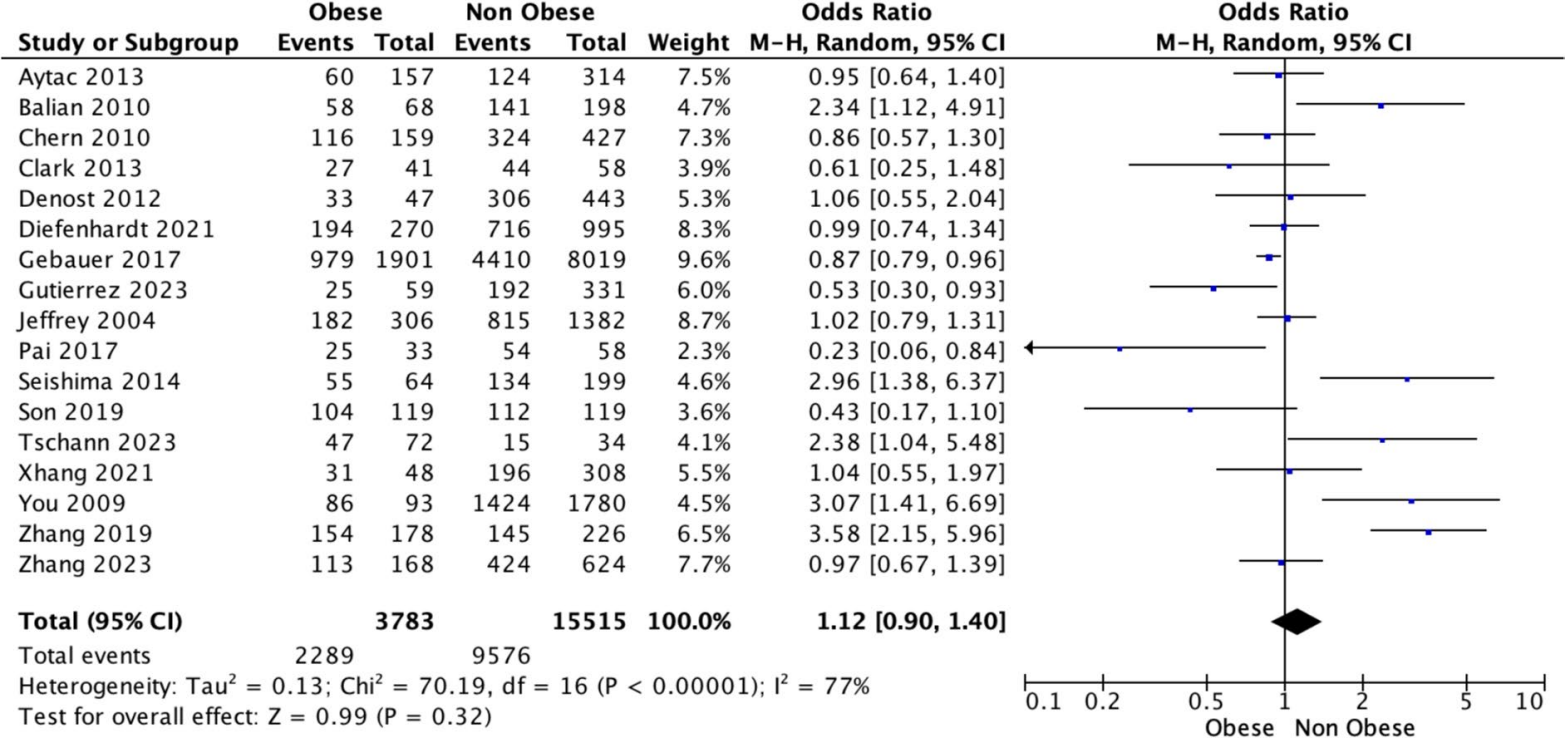
Forest plot demonstrating disease-free survival for patients being treated for rectal cancer living with and without obesity using the Mantel–Haenszel method

**Fig. 3:**
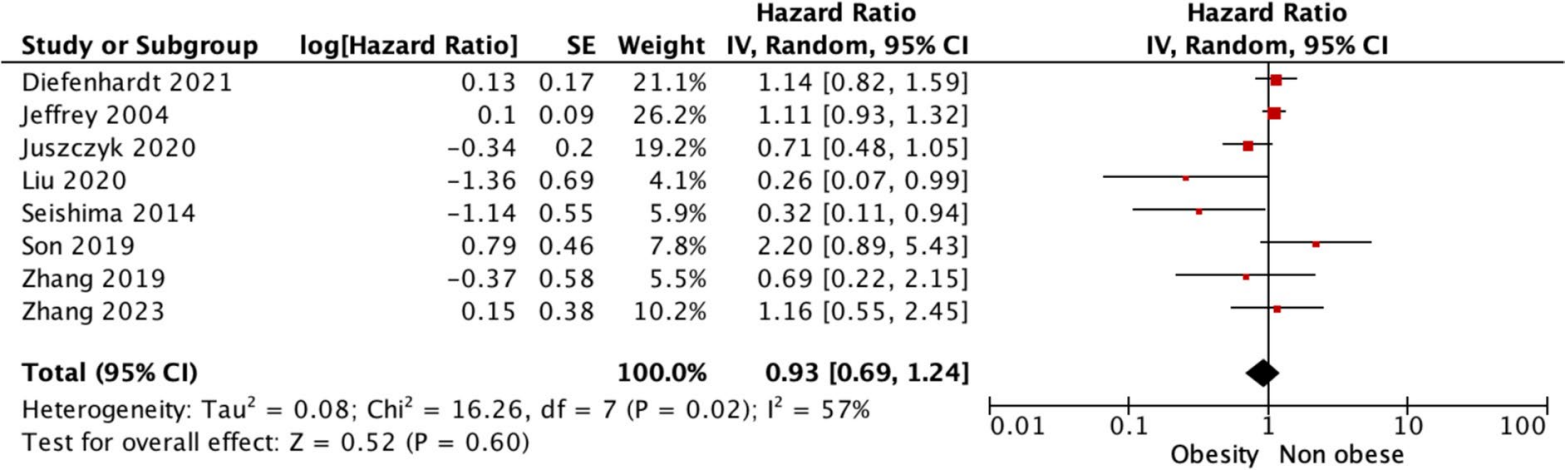
Forest plot demonstrating disease-free survival for those patients being treated for rectal cancer with and without obesity using time-to-effect modelling using the generic inverse variance method

Overall, 18 studies reported DFS outcomes for those living with and without obesity being treated for rectal cancer. There was a significant difference observed in the estimated DFS in favour of those living without obesity relative to their counterparts living with obesity [62.4% (9576/15,515) vs. 60.5% (2289/3783), *P* = 0.029, †] (Table 2). At meta-analysis, estimated DFS were similar for those living with or without obesity [OR 1.12, 95% CI 0.90–1.40, *P* = 0.320, heterogeneity (*I*^2^) = 77%] (Fig. 2). Using time-to-effect modelling, estimated DFS were similar for those living with or without obesity [HR 0.93, 95% CI 0.69–1.24, *P* = 0.600, *I*^2^ = 57%] (Fig. 3).

**Table 2:** Study specific data comparing the association between survival outcomes for those living with and without obesity being treated with curative intent for rectal cancer

Title	Year	DFS obesity	DFS non-obese	OS obesity	OS non-obese
Aytac	2013	60/157	124/314	67/157	134/314
Baird	2019	-	-	38/42	149/178
Balian	2010	58/68	141/198	63/68	158/198
Chern	2010	116/159	324/427	143/159	359/427
Clark	2013	27/41	44/58	29/41	49/58
Denost	2012	33/47	306/443	41/47	363/443
Diefenhardt	2021	194/270	716/995	-	-
Gebauer	2017	979/1901	4410/8019	1165/1901	4010/8019
Gutierrez	2023	25/59	192/331	35/59	199/331
Jeffrey	2004	182/306	815/1382	169/306	746/1382
Juszczyk	2020	-	-	101/131	238/322
Kalb	2019	-	-	108/127	398/485
Liu	2020	-	-	-	-
Pai	2017	25/33	54/58	27/33	55/58
Seishima	2014	55/64	134/199	-	-
Son	2019	104/119	112/119	109/119	104/119
Soo Han	2020	-	-	33/42	1013/1384
Sun	2017	-	-	49/61	204/249
Tschann	2023	47/72	15/34	-	-
Xhang	2021	31/48	196/308	32/48	206/308
You	2009	86/93	1424/1780	-	-
Zhang	2023	154/178	145/226	155/178	158/226
Zhang	2019	113/168	424/624	136/168	505/624
		60.5% (2289/3783)	62.4% (9576/15,515)	67.8% (2500/3687)	59.8% (9048/15,125)
		*P* = 0.029, †		*P* < 0.001, †	

*OS* overall survival, *DFS* disease-free survival

^†^denotes Fisher’s Exact test

### Overall survival

**Fig. 4:**
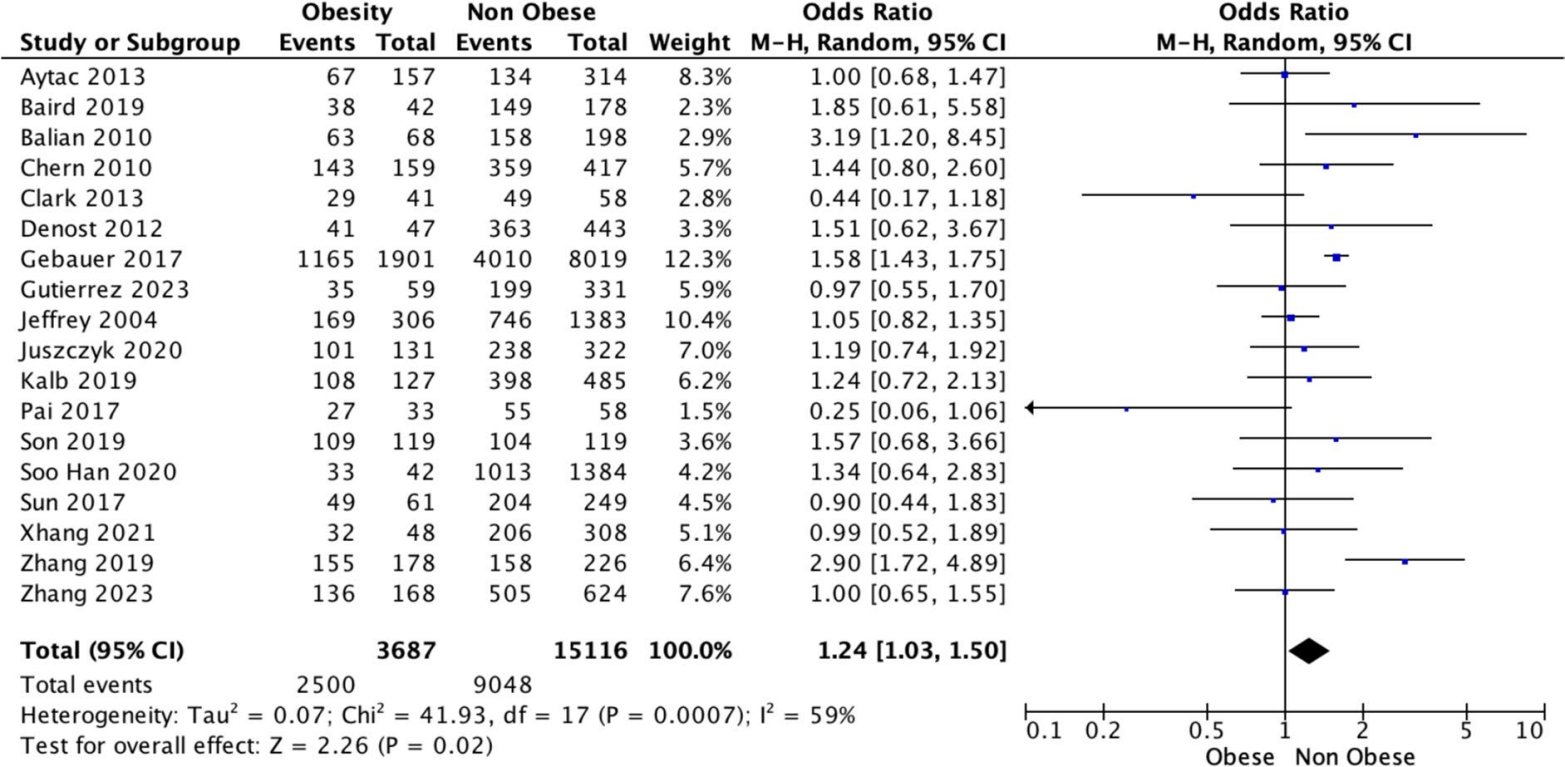
Forest plot demonstrating overall survival for patients being treated for rectal cancer living with and without obesity using the Mantel–Haenszel method

**Fig. 5:**
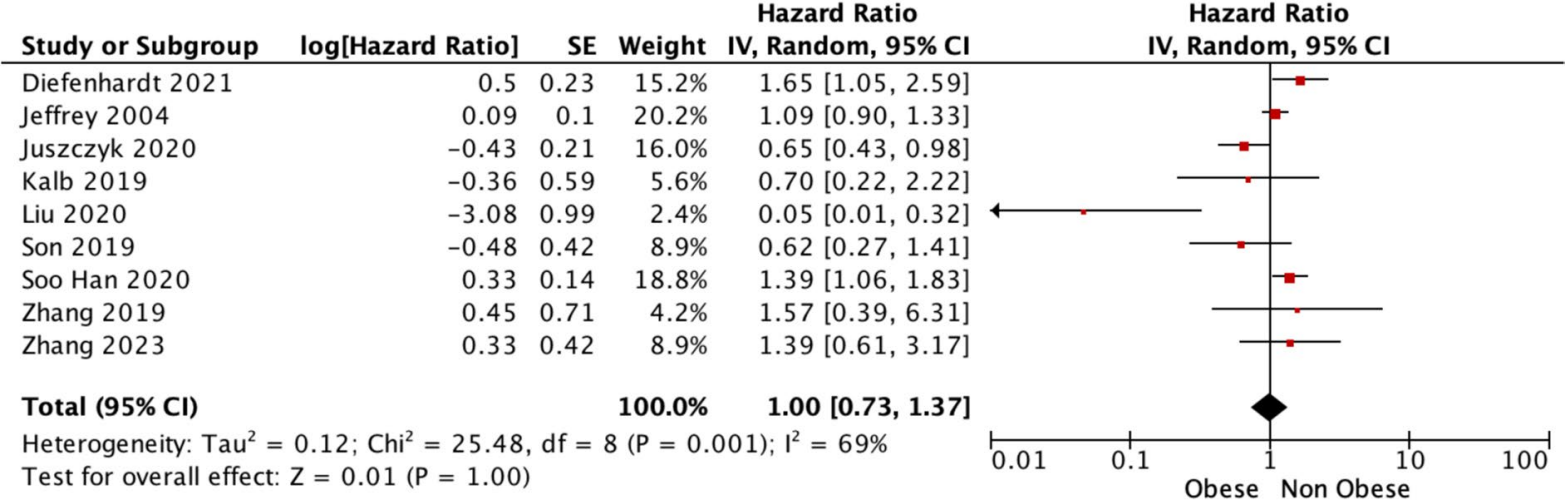
Forest plot demonstrating overall survival for patients being treated for rectal cancer living with and without obesity using time-to-effect modelling using the generic inverse variance method

Overall, 20 studies reported OS outcomes for those living with and without obesity being treated for rectal cancer. There was a significant difference observed in the estimated OS in favour of those living with obesity relative to their counterparts living without obesity [67.8% (2500/3687) vs. 59.8% (9048/15,125), *P* < 0.001, †] (Table 2). At meta-analysis, estimated OS were favourable for those living with obesity relative to those living without obesity [OR 1.24, 95% CI 1.03–1.50, *P* = 0.020, (*I*^2^) = 59%] (Fig. 4). Using time-to-effect modelling, estimated OS were similar for those living with or without obesity [HR 1.00, 95% CI 0.73–1.37, *P* = 1.000, *I*^2^ = 69%] (Fig. 5).

## Discussion

To the authors’ knowledge, this is the first systematic review and meta-analysis performed to decipher the implications of obesity on the oncological and survival outcomes of patients being treated surgically with curative intent for primary rectal cancer. This study included 22,250 patients from 23 studies, and the most important finding was the results which challenge previous connotations suggesting that an ‘obesity paradox’ exists and is beneficial for this patient cohort. Importantly, previous meta-analyses extrapolated that patients with CRC were protected by this theoretical paradox; however, this theory has been successfully challenged by the results of this study, through indication that those living with obesity are as likely to suffer poor oncological outcomes as their counterparts. These are clinically relevant findings, which warrant consideration by the colorectal multidisciplinary team during perioperative counselling.

The current study presents raw data demonstrating superior OS in favour of those living with obesity (67.8% vs. 59.8%, *P* < 0.001). This is then ratified in the meta-analysis, when the Mantel–Haenszel method was deployed [OR 1.24, 95% CI 1.03–1.50, *P* = 0.020]. Subsequently, and more appropriately, the adoption of time-to-effect proportional hazards modelling led to results which refuted the findings of these more primitive analyses; in this study, patients living with obesity being treated for rectal cancer failed to outperform their counterparts [HR 1.00, 95% CI 0.73–1.37, *P* = 1.000]. It is imperative to note that there are important statistical nuances which must be respected when applying such analyses to clinical data. Importantly, the power of time-to-event models should be considered in the realm of survival analyses, in particular, in scenarios when the effect of exposures is analysed over a continuous timeframe [43]. Mantel–Haenszel testing (in the absence of log-rank testing, as deployed here) involves the adaptation of a stratified 2 × 2 table which delivers odds ratios, where events are observed and encapsulated in a discrete time period [44, 45]. Accordingly, their utility for deciphering the cause-effect of medical comorbidities (such as obesity) upon survival outcome is not recommended [46]. With this in mind, one must be cognisant of the importance of refraining from ‘chasing’ statistical significance in such instances, by ensuring the most relevant and appropriate statistical methods are utilised for clinical research studies [47]. Accordingly, the conclusions of this study can be justified, as the theory that an ‘obesity paradox’ truly is responsible for improving survival outcomes in those being treated for rectal cancer is not apparent based on these data. Furthermore, this study supports perceptions that this concept is ‘highly nuanced’, which is likely due to several complex metabolic, immunogenic and environmental factors which confound this theory [48].

Interestingly, these results are of particular relevance given their context for the surveillance window following successful treatment for primary rectal cancer: The follow-up period of 59.7 months in this study accurately reflects the recommended 5-year surveillance period, as outlined by the American Society of Colon and Rectal Surgeons (ASCRS) in their guidelines [49]. Therefore, once patients are considered in remission of their cancer, it is important that clinicians hold themselves responsible for increasing patient awareness of the importance of optimising BMI and other metabolic parameters. In essence, this study demonstrates that there is limited premise to believe that increased BMI is important in improving oncological and survival outcomes in this cohort. Therefore, there should be no ambiguity with respect to the message from this study—patients in remission should be encouraged to achieve a healthy BMI if possible, as no meaningful survival advantage can be ascertained through an ‘obesity paradox’ based on the data derived in this study.

The study is subject to certain limitations. Firstly, this study includes patient data which is largely from observational studies, most typically of retrospective design; studies of such design are unfortunately exposed to selection, confounding and ascertainment biases. This limits the data utilised in this study. Secondly, while 18.5% of patients in this study were reported to be living with obesity at the time of cancer diagnosis, there is no data to demonstrate how their malignancy, cancer therapeutics, or surgical management impacted upon patient their BMI after diagnosis and treatment. In essences, this is a ‘once off’ measurement of BMI which is then extrapolated to represent the patients BMI for the entirety of follow-up within these studies. It has been well described that patient body weight changes significantly during cancer treatment [50] for several reasons, including tumour necrosis factor (TNF, or cachexin), a cytokine released from tumour cells which is responsible for tumourgenesis, while also having a profound effect on appetite, suppressing hunger considerably leading to weight loss in the cancer patient [51]. Finally, the data presented in this study comes from studies from various countries across the world, with significant heterogeneity anticipated in their diet consumption, cultural exposures, and medical practice. Thus, the transferability of these results to individual patients may be challenging and cautious interpretation by the primary physician may be warranted.

In conclusion, once diagnosed and being treated with curative intent for rectal cancer, patients living with obesity exhibit similar survival outcomes as those living without obesity. This study refutes hypotheses that an ‘obesity paradox’ is protective for survival in patients with rectal cancer. Given the current obesity epidemic, this concept may warrant incorporation into preoperative counselling by the multidisciplinary team providing care to patients with rectal cancer.

## Data Availability

Data can be made available upon reasonable request from the corresponding author.
